# Serum calcitonin negative Medullary thyroid carcinoma

**DOI:** 10.1186/1477-7819-4-97

**Published:** 2006-12-21

**Authors:** Michael Sand, Marcos Gelos, Daniel Sand, Falk G Bechara, Gerd Bonhag, Ellen Welsing, Benno Mann

**Affiliations:** 1 Department of General and Visceral Surgery, Augusta Krankenanstalt, Academic Teaching Hospital, Ruhr-University Bochum, Germany; 2 Department of Physiological Science, University of California Los Angeles (UCLA), California, USA; 3 Department of Dermatology and Allergology, Ruhr-University Bochum, Germany

## Abstract

**Background:**

Medullary thyroid carcinomas (MTC) constitute about 5 to 7 % of thyroid neoplasms. They originate from parafollicular C cells which produce Calcitonin, a hormone which has an impact on calcium metabolism and represents the biochemical activity of MTC. In rare cases pre-operative serum calcitonin can be negative.

**Case presentation:**

We report on a 73-year-old female patient with a rare case of a serum calcitonin negative medullary thyroid carcinoma who suffered fulminant post-operative course and died of multiple metastasis.

**Conclusion:**

This case shows that in very rare cases MTCs do not secrete calcitonin making diagnosis and tumour follow-up difficult. To this date, only few reports describing this combination of circumstances were found in the English literature.

## Background

Medullary thyroid carcinomas (MTC) constitute about 5 to 7 % of thyroid neoplasms. They originate from parafollicular C cells which are derived from the ultimobranchial body and neural crest [1]. C cells produce Calcitonin, a hormone which has an impact on calcium metabolism. Microscopically the tumor is characterized by small nests of small, round cells, production of amyloid and dense irregular areas of calcification [1]. Cervical lymph node metastases occur early in the disease in about 50 % of cases with distant metastases occurring later. Cervical metastases most commonly involve the central, paratracheal and jugular regions. Distant metastases favor the mediastinum, liver, lung and bone [2]. The incidence in male/female ratio is roughly equal over a wide age range. Most of MTC are sporadic and usually unilateral. Familial medullary carcinoma occurs in the multiple endocrine neoplasia syndromes (MEN) of the type 2 variety. When occurring in association with MEN syndromes it is usually bilateral and typically the first abnormality observed in both MEN 2A and 2B syndromes.

The biochemical activity of medullary thyroid carcinoma includes production of calcitonin and cacinoembryogenic antigen (CEA). In rare cases pre-operative serum calcitonin can be negative. To this date, only few reports describing this combination of circumstances were found in the English literature [3-5].

## Case presentation

**Figure 1:**
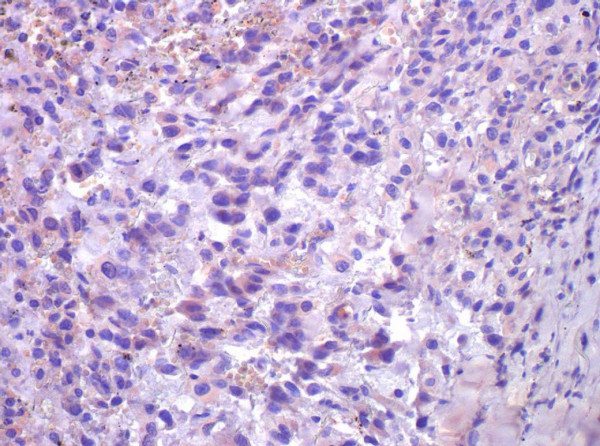
Histopathology showing a MTC with weak positive immunhistochemistry for Calcitonin (× 40).

**Figure 2:**
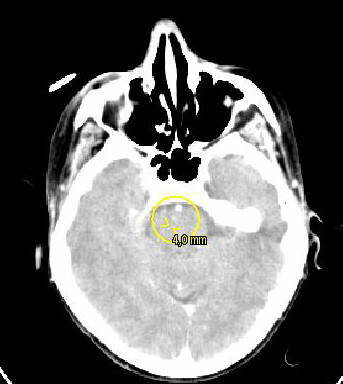
Horizontal section of the head (CT-scan) showing an intracerebral metastasis (diameter 0,4 mm).

**Figure 3:**
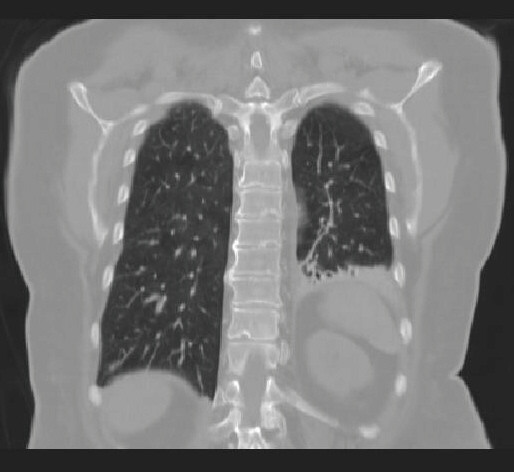
Coronal section of the chest (CT-scan) showing multiple lung metastases.

We report on a 73-year-old female patient with an 8-year history of thyroid nodules for which she underwent regular clinical examination and blood tests. Three weeks before referral to our endocrinological surgery unit she developed a swelling of her head and neck lymph nodes. Her past medical history included surgical removal of a benign breast tumor 22 years ago. Her family history was unremarkable regarding any endocrinological or thyroid disease. Besides her obesity she was suffering from reflux disease and hypertension. She had a regular pulse of 68/per minute and blood pressure of 120/70 mmHg under antihypertensive medication. Her muscle tone was reduced. She had observed a change of her voice and had lost 20 kg of weight within the past 4 months. A thyroid scintigram showed multiple confluencing cold nodules which were highly suspicious for malignant neoplasm of both thyroid lobes and regional lymph node metastases along both jugular veins. Clinical exam and sonography showed multiple enlarged lymph nodes of the head and neck. Pre-operative serum calcitonin was 5.3 pg/ml (normal range 0.8–9.9 pg/ml). Based on the history, clinical presentation and the scintigram we suspected an anaplastic thyroid carcinoma and performed a radical oncologic thyroidectomy with bilateral modified neck-dissection and resection of the right internal jugular vein and the left recurrent laryngeal nerve. Intraoperatively the tumor was tightly adherent to the surface of the trachea. Because of the impressing clinical picture a fine needle aspiration was not performed prior to surgery. In the authors' point of view it would have had no prognostic effect at this advanced stage of disease. A pre-operative pentagastrin test was also not performed as clinical presentation made the presence of an anaplastic carcinoma highly suspicious. Surprisingly, histopathology showed a pT3 L1 V1 R1 N1b low-differentiated medullary-thyroid carcinoma with massive lymphangiosis and haemangiosis carcinomatosa. Immunhistochemistry revealed weak positive staining for calcitonin and carcinoembryogenic antigen (CEA) (Figure 1). Post-operative serum calcitonin decreased to 2.47 pg/ml still being within normal range (0.8–9.9 pg/ml). Post operatively the patient was in the intensive care unit for 6 days. A pentagastrin stimulation test was not performed due to the bad condition of the patient. A permanent tracheostoma was established to ensure a patent airway as the patient aspirated after initial extubation. Additionally massive crust formation obstructed the patient's airway which was treated with a humidifier and frequent irrigations with physiological saline solution and subsequent suctioning of the trachea. Unfortunately the patient developed central lung emboli in the right pulmonary artery. A postoperative CT scan showed multiple lung and intracerebral metastases (Figure 2 and Figure 3). The patient died six weeks after diagnosis.

## Discussion

Medullary thyroid carcinoma is a rare calcitonin-secreting neoplasm that occurs in both a familial and sporadic form [6]. It is the most aggressive well differentiated thyroid carcinoma, with survival rates of 40–50 % at 10 years [7]. Routine measurements of serum calcitonin levels are considered to be an integral part of the diagnostic evaluation of thyroid nodules and the diagnosis of medullary thyroid carcinoma. It has been suggested that calcitonin testing, both with and without pentagastrin stimulation, may facilitate early diagnosis of this disease and ultimately decrease morbidity and mortality [8-12]. Serum CT is the most specific and sensitive marker of MTC for both the primary diagnosis and the postsurgical follow-up [13]; it is produced in abnormally high concentrations by almost 100 % of primary and metastatic MTCs. However in rare cases, as described here, serum calcitonin can be negative. Although our patient had a voluminous, rapidly growing medullary thyroid carcinoma with multiple metastases pre-operative serum calcitonin was within normal range. An calcitonin assay-specific problem is unlikely because different assays were used postoperatively (Nicols Advantage Chemiluminescence Assay, Nichols Institute Diagnostics Inc., San Clemente, CA, USA and Immulite Calcitonin Assay, DPC-Bühlmann GmbH, Salzburg, Austria). Before referral to our department the patient's thyroid nodules were monitored by annual checks of thyroid hormone and serum calcitonin. One would expect that the expression of an antigen in the neoplastic cells would be associated with the hypersecretion of the corresponding antigen in the circulation, as in the case of Calcitonin and CEA. The levels of these antigens are described to be elevated in patients with metastastatic medullary thyroid carcinoma and as accurate reflections of new metastases [12-15]. Regrettably this was not the case in our patient. It is possible that rare forms of MTC go along with mutations in the calcitonin/CGRP gene which might be responsible for the normal or relatively reduced calcitonin levels. It would be interesting to examine the calcitonin/CGRP gene structure in these patients. Analysis of white blood cell DNA by Southern blot hybridizations could show deletions or rearrangements in the calcitonin/CGRP gene locus. Additionally defects in the cellular machinery needed for calcitonin synthesis and/or secretion could be responsible. The tumour showed positivity for calcitonin in the immunhistochemistry which is in line with the latter hypothesis that calcitonin production is active but secretion mechanisms might be altered. Bockhorn et al. also reported a similar case of a 50 year old woman with a serum calcitonin negative MTC. Comparing the histologies we have observed some (uncharacteristic) similarities: the cells described were also relatively large with an increased caryoplasmic ratio, indistinct cell borders and partly spindled cytoplasm. It would be interesting to compare the histologies of other serum calcitonin negative MTC's which might show histomorphologic similarities.

This report illustrates that in rare cases the diagnosis of medullary thyroid carcinoma cannot be excluded by means of laboratory values such as serum calcitonin. When serum calcitonin is negative a cytomorphologic analysis of fine needle aspiration and a determination of RET proto-oncogene mutations are two diagnostic tools which are available for detection and accurate management of patients in whom medullary thyroid carcinoma is expected [16].

## Conclusion

This case shows that in very rare cases MTCs do not secrete calcitonin making diagnosis and tumour follow-up difficult.

## Conflict of interests

All authors hereby disclose any commercial associations which might pose or create a conflict of interest with information presented in this manuscript. All authors declare that they have no competing interests.

## Authors' contributions

**MS**: documented and prepared the draft

**MG**: Revised and edited most of the manuscript and helped in preparing the draft

**DS**: Literature search, revision of bibliography and helped with editing of the manuscript

**FGB**: Literature search and edited part of the manuscript

**EW**: Surgeon who performed the operations and helped in preparing the manuscript

**GB**: Surgeon who performed the operations and helped in preparing the manuscript

**BM**: Surgeon who performed the operations and edited part of the manuscript and helped in preparing the draft

All authors read and approved final manuscript.
